# A Complex Ventricular Septal Defect Causing Severe Aortic Insufficiency

**DOI:** 10.7759/cureus.12532

**Published:** 2021-01-06

**Authors:** Monique Oye, Dominika Zoltowska, Dheeraj Gopireddy, Robert Percy, Srinivasan Sattiraju

**Affiliations:** 1 Medicine, University of Florida College of Medicine – Jacksonville, Jacksonville, USA; 2 Cardiology, University of Florida College of Medicine – Jacksonville, Jacksonville, USA; 3 Radiology, University of Florida College of Medicine – Jacksonville, Jacksonville, USA

**Keywords:** vsd, echocardiogram, cardiomyopathy, aortic insufficiency

## Abstract

We report a case of a 60-year-old male with decompensated heart failure secondary to severe aortic insufficiency in the setting of a complex ventricular septal defect. The case highlights the use of multimodality imaging, including transthoracic echocardiogram, transesophageal echocardiogram, and cardiac magnetic resonance imaging, which contributed to the findings and diagnosis of the defect noted and was confirmed during surgery. The images provide an exceptional understanding of a complex ventricular septal defect and the associated pathology, which resulted in severe aortic regurgitation leading to cardiomyopathy. Although traditionally ventricular septal defects can be classified into certain types, our case highlights that some of these defects are very complex and require multimodality imaging for evaluation.

## Introduction

Ventricular septal defects (VSD) are the most common congenital cardiac malformations. They occur in less than 5% of live births, and a significant percentage spontaneously close prior to adulthood [1]. VSDs are generally classified by anatomic location, specifically whether they are located above or below the crista supraventricularis. Those located below are called subaortic, perimembranous, and muscular defects. Those located above are called supracristal VSDs and account for only 2-3% of all VSDs [2]. More commonly seen in patients of Eastern Asian descent, supracristal VSDs often cause aortic insufficiency due to associated prolapse of the aortic valve. In such cases, surgical closure of the VSD and suspension of the aortic valve is most commonly indicated [3]. Our patient had a complex VSD that was evaluated using multi-modality imaging.

## Case presentation

A 60-year-old African-American man with a history of a VSD and non-ischemic cardiomyopathy (New York Heart Association [NYHA] Class III) presented to the hospital with complaints of worsening dyspnea and twenty-pound weight gain over three months. Physical exam revealed that he was significantly volume overloaded with bi-basilar crackles auscultated on lung exam and 3+ pitting edema up to his bilateral thighs. A grade 4/6 systolic murmur was heard at the left lower sternal border, and a grade 3/6 diastolic murmur was heard in the right upper sternal border. He was admitted for acute chronic decompensated heart failure requiring aggressive diuresis.

**Figure 1:**
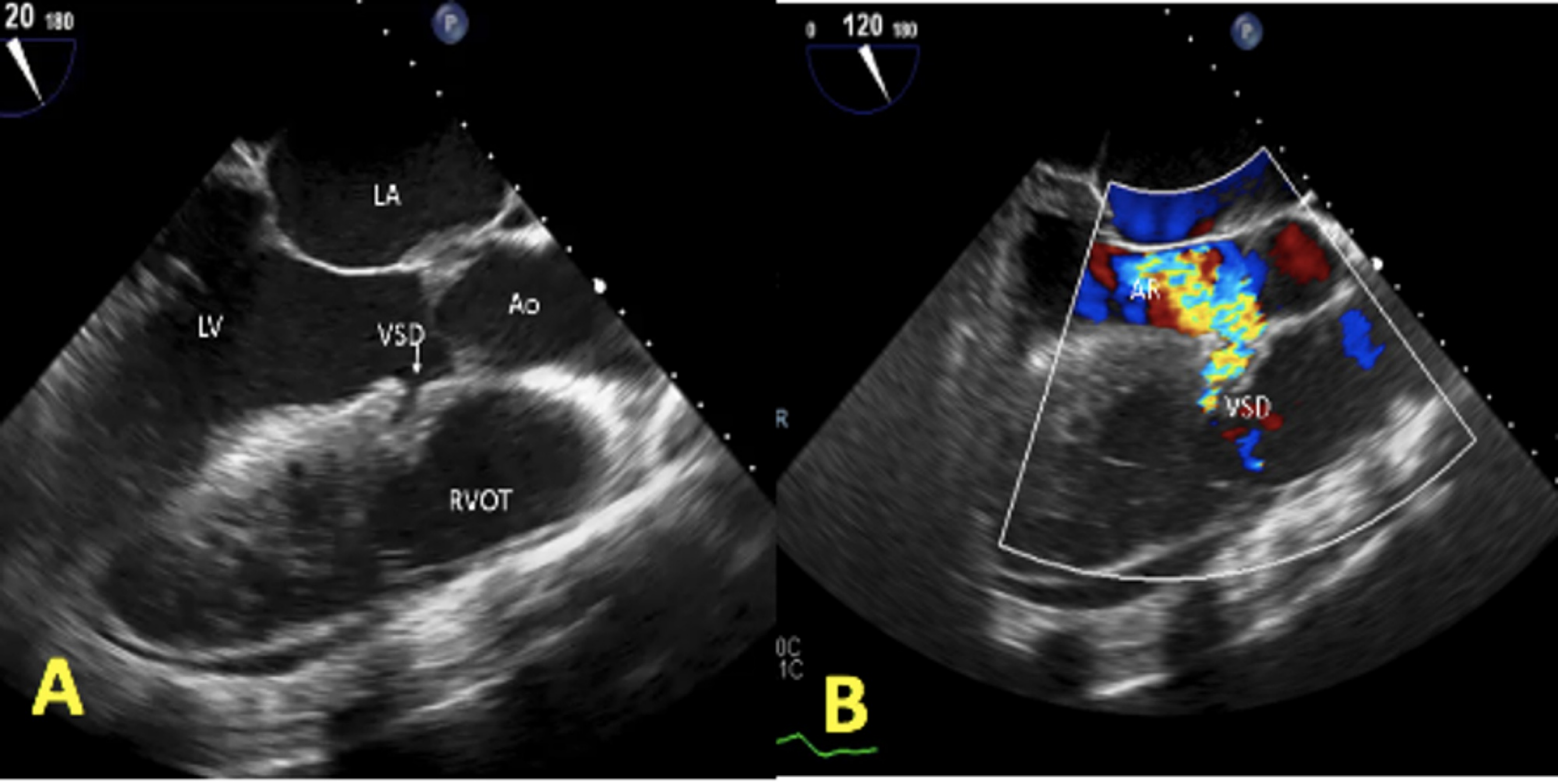
Two-dimensional transesophageal echocardiogram (TEE) images showing the small ventricular septal defect (VSD) in the upper part of the septum with a left to right shunt (A), and severe aortic insufficiency (B)

**Figure 2:**
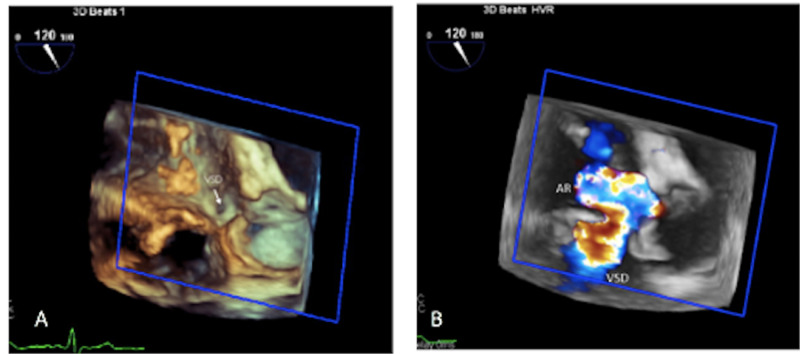
Three-dimensional transesophageal echocardiogram (TEE) image showing the ventricular septal defect (VSD) opening below right coronary cusp (A), and severe aortic insufficiency with flow through VSD with a left to right shunt

**Figure 3:**
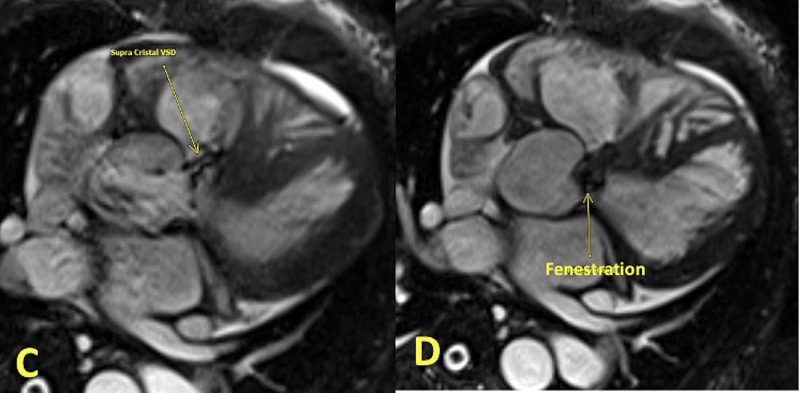
Axial three-chamber balanced steady-state free frecession (bSSFP) sequence Axial three-chamber balanced steady-state free frecession (bSSFP) sequence demonstrates a small defect in the supracristal region (arrow) communicating with the right ventricle (C) and a small defect in the posterior cusp of the aortic valve (arrow). Small regurgitating jets were seen on the cine images (not shown here) compatible with the fenestration of the valve (D).

A transthoracic echocardiogram (TTE) was performed, which showed a dilated left ventricle with an ejection fraction (EF) of 40%, a small ventricular septal defect (VSD) in the upper part of the septum with a left to right shunt, and severe aortic insufficiency (AI). These findings were further evaluated with a transesophageal echocardiogram (TEE), which revealed a VSD located right below the right coronary cusp suggestive of a probable supracristal VSD. However, this VSD had an irregular track extending through the basal septum into the right ventricular outflow tract (RVOT), causing a left to right shunt. In addition, severe aortic regurgitation was noted (Figures 1-2). Cardiac magnetic resonance imaging (MRI) was pursued for precise delineation of VSD and aortic valve anatomy (Figure 3). It was clarified that the VSD is located in the supracristal septum under the right aortic cusp causing prolapse, leading to severe aortic regurgitation (Videos 1-2). Interestingly, cardiac MRI also visualized aortic leaflets fenestration contributing to aortic insufficiency.

**Video 1:** 2D color showing flow through VSD and severe AI VSD - ventricular septal defect; AI - aortic insufficiency

**Video 2:** Severe aortic insufficiency and flow through VSD with a left to right shunt VSD - ventricular septal defect

Due to the location of the VSD and concurrent severe aortic valve dysfunction, the patient was not deemed as a suitable candidate for percutaneous closure. Preoperative right and left heart catheterization was performed, which showed normal coronary arteries and mild pulmonary hypertension. The patient underwent surgical repair of VSD with a pericardial patch. Intraoperative surgical evaluation of the aortic valve confirmed the localization of the VSD and the presence of aortic leaflets fenestration. Aortic valve repair was attempted; however, there was residual moderate aortic regurgitation. The patient required aortic valve replacement with a 25 mm Medtronic Mosaic® tissue (Medtronic, Inc., Minneapolis, USA). Intra procedural TEE confirmed no flow across the patch that closed VSD and well-functioning aortic prosthesis. 

On outpatient follow-up two weeks later, he endorsed reduced dyspnea and improved exercise tolerance with NYHA Class II functional status. Follow-up TEE in two months showed no residual leak of the VSD patch and well-functioning bioprosthetic valve.

## Discussion

This is a unique case of complex ventricular septal defect accompanied by aortic valve fenestration, emphasizing the important role of multimodality imaging. Supracristal VSD is a rare anatomical form of septal defect above the crista supraventricularis, accounting for only 2-3% of all VSDs [2]. Sporadically, the defect may extend to a muscular portion of the septum. Though commonly diagnosed and corrected at an earlier age [3], uncorrected supracristal VSDs often present with decompensated heart failure symptoms and may result in concomitant aortic insufficiency due to prolapse of the aortic valve, most commonly the right coronary cusp, from the venturi effect of the left to right shunt [4]. We hypothesize that the right coronary cusp suffered thinning and probably developed fenestration due to the impact of the jet of aortic regurgitation. Echocardiography remains the first-line diagnostic modality. Nevertheless, a complete evaluation of congenital disease often requires the use of advanced cardiac imaging. The precise anatomy delineation is crucial while planning further management. Definitive treatment involves endovascular or surgical closure of the VSD and may require concurrent surgical replacement of the aortic valve [5], as in our case. 

## Conclusions

TTE, TEE, and cardiac MRI are useful in the evaluation of VSD. These imaging modalities may be complementary in providing diagnostic information regarding the defect. VSDs can result in significant aortic regurgitation due to prolapse of aortic cusps. This may lead to left ventricular dysfunction.
